# An Atypical Case of Superior Vena Cava Syndrome Due to Sarcomatoid Lung Cancer With Pericardial Metastasis

**DOI:** 10.14740/jmc5293

**Published:** 2026-04-29

**Authors:** Aryan Patel, Linda Akbarshahi, Jotrineesha L. Walton, Hardeep Singh

**Affiliations:** a GME Research, Northeast Georgia Medical Center, Gainesville, GA, USA; b Family Medicine, Northeast Georgia Medical Center, Gainesville, GA, USA

**Keywords:** Superior vena cava syndrome, Lung cancer, NSCLC

## Abstract

Superior vena cava (SVC) syndrome is commonly caused by malignancies from lung cancers, thrombus, and indwelling intravascular devices. Specifically, SVC syndrome is mostly associated with malignancies such as small cell lung cancer (SCLC) and non-small cell lung cancer (NSCLC). Pulmonary sarcomatoid carcinoma (PSC), as a rare type of NSCLC, accounts for 0.1% to 0.4% of pulmonary tumors. PSC’s rapid progression, aggressive growth, and complexity to diagnose help differentiate from other thoracic malignancies, yet due to its rarity, there is limited literature documenting an association between this entity and SVC syndrome. Here we present an atypical case of SVC syndrome with a history of tobacco use and chronic right upper extremity lymphedema who developed facial swelling, dyspnea, and worsening right upper extremity swelling. Imaging revealed a large mediastinal mass compressing both the SVC and pulmonary artery. Further workup and endobronchial ultrasound (EBUS) were performed, which led to a diagnosis of poorly differentiated sarcomatoid carcinoma. Additionally, pericardiocentesis revealed features consistent with adenocarcinoma. The case was further complicated with hypoxic respiratory failure and new-onset atrial fibrillation. With extensive intensive care unit (ICU) care, the patient was discharged with a medication regiment, and referred to oncology, cardiology, and palliative care. Patient declined chemotherapy and immunotherapy, opting for hospice care. Patient stay was complicated by hypoxia respiratory failure, pleural effusions, pericardial effusion, and atrial fibrillation with rapid ventricular response (RVR). This case emphasizes that SVC syndrome can be associated with poorly differentiated sarcomatoid carcinoma.

## Introduction

Superior vena cava (SVC) syndrome is an uncommon medical condition with an estimated 15,000 cases reported each year in the United States [1], and incidence rates as uncommon as one in 3,100 patients [2]. It usually presents with face/neck swelling, distended neck veins, cough, dyspnea, orthopnea, upper extremity swelling, and conjunctival suffusion; however, less common symptoms can be stridor, pleural effusion, syncope, cyanosis, stupor, and coma [2]. It commonly occurs due to a partial or complete obstruction of the SVC by an external compression (a tumor), internal occlusion (thrombus), or even indwelling intravascular devices: catheters, pacemakers, and implantable cardioverter-defibrillator (ICD) [3]. SVC syndrome is initially screened through ultrasounds of the jugular, subclavian, and innominate veins, yet most confirmed diagnoses are obtained through contrast-enhanced computed tomography (CT) scans, digital subtraction venography, and magnetic resonance venography (MRV) scans [3]. Clinically, treatment plans comprise removal of intravascular devices (with the addition of anticoagulation therapy and catheter-directed thrombolysis), chemotherapy, radiation, and most commonly endovascular repair [2]. Common comorbidities associated with SVC syndrome include several types of lung cancers (diffuse large B-cell lymphoma, lymphoblastic lymphoma, and primary mediastinal large B-cell lymphoma [4]), vasculitis [5], diabetes [6], and heart failure [7]. Specifically, lung cancer has been seen to be one of the leading causes of SVC syndrome, with 60–85% of cases correlated with this illness [8]. Here, we present an atypical case of a poorly differentiated sarcomatoid carcinoma with central mediastinal invasion that is likely causing SVC syndrome, with uncommon comorbidities: pericardial effusion and new-onset atrial fibrillation.

## Case Report

A 61-year-old male truck driver with a history of tobacco use (60–80 packs per year), alcohol use (roughly 10 drinks per week), chronic right upper extremity lymphedema, childhood asthma, chronic supraventricular tachycardia (SVTs), prior deep vein thrombosis (DVTs) previously treated with apixaban (discontinued due to allergic reaction; currently on aspirin), and pericardial effusion presented to the emergency department with worsening right upper extremity swelling, progressive dyspnea, and facial swelling.

**Figure 1:**
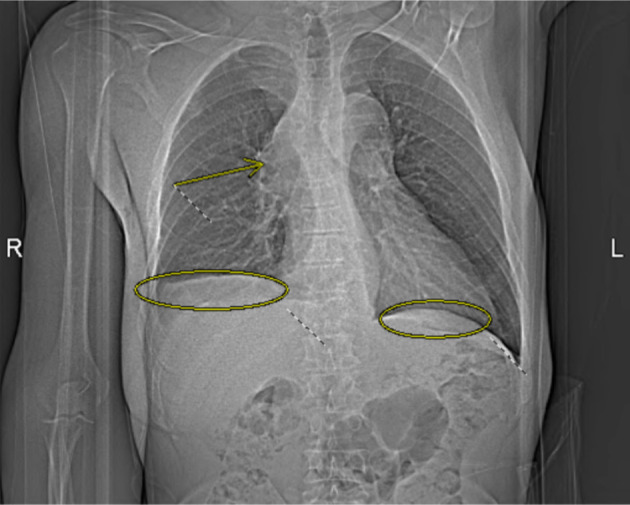
Computed tomography angiography (CTA) pulmonary chest X-ray obtained on the day of admission demonstrates bilateral pleural effusions (circled) and a mediastinal mass causing severe superior vena cava (SVC) compression and right upper lobe pulmonary artery obstruction (arrow).

Upon physical examination, the patient appeared to have distended upper chest veins, bilateral upper extremity swelling (more on right side), rales (oxygen saturation was 99% on room air), and mild facial swelling. Lab results displayed the patient being anemic with a hemoglobin of 11.4 g/dL (normal: 14.0–17.5 g/dL), elevated white blood cell count (WBC) level of 13.8 cells/µL (normal: 4.0–11.0 × 10^3^/µL), eosinophils of 11% (normal: 0–5%), and an elevated platelet count of 447,000/µL (normal: 150–400 × 10^3^/µL). The differential diagnosis included: DVT of the upper extremity, SVC syndrome, lymphedema exacerbation, primary lung cancer, lymphoma, and infection. In view of the patient’s history of DVT, an initial duplex ultrasound was ordered to rule out acute DVTs. Furthermore, a computed tomography angiography (CTA) pulmonary series was conducted which revealed bilateral pleural effusions, moderate pericardial effusions, and a large mediastinal mass causing severe SVC compression and right upper lobe pulmonary artery obstruction (Fig. 1). Notably, patient was also seen in the emergency department a few months prior, and chest X-ray revealed no abnormalities at the time.

At the time, the patient was treated with heparin for venous thromboembolism (VTE) prophylaxis, ampicillin/sulbactam and azithromycin for possible community-acquired pneumonia (CAP), prednisone for possible COPD exacerbation, and supplemental oxygen via nasal cannula. Due to severity of SVC compression and differential diagnosis of primary lung malignancy versus metastatic disease, radiation oncology was consulted for tissue diagnosis. Yet due to the patient’s respiratory distress, an emergent palliative radiation therapy was ordered to reduce the mass, and the patient was admitted to family medicine.

**Figure 2:**
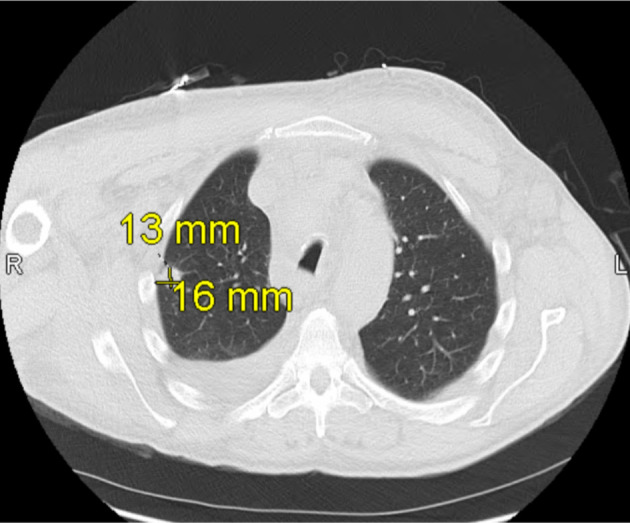
Computed tomography (CT) scan of the chest obtained on hospital day 2 shows a 13 × 16 mm mass in the lateral right upper lobe with irregular margins and extension to the pleura, consistent with findings during early hospitalization for suspected superior vena cava (SVC) syndrome.

**Figure 3:**
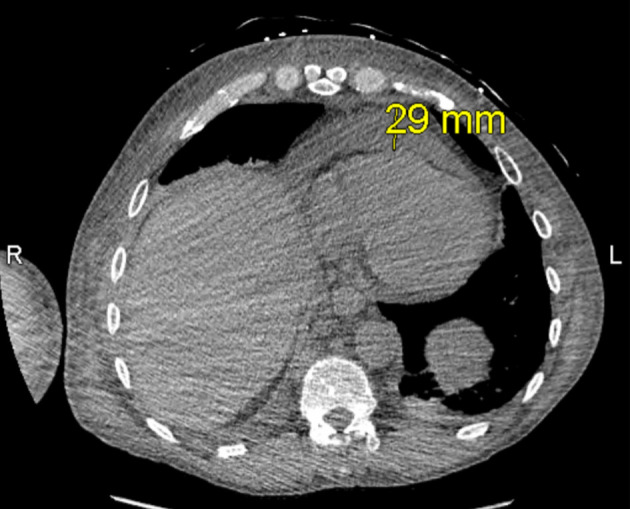
Computed tomography (CT) ion scan of the chest obtained on hospital day 4, depicting pericardial effusion.

Once admitted, cardiothoracic surgery was consulted and concluded that there were no surgical options for the patient. As the patient’s symptoms progressed, a thoracentesis was performed as well as the initiation of radiation therapy to help reduce the mediastinal mass for suspected SVC syndrome. Additional imaging revealed a 13 × 16 mm mass laterally in the right upper lobe with irregular margins and extension to the pleura laterally (Fig. 2), increase in right pleural effusion, decrease in left pleural effusion, increase in pericardial effusion (Fig. 3), and right upper lobe ground-glass opacities, consistent with pneumonia or atelectasis.

An endobronchial ultrasound (EBUS) procedure with bronchoalveolar lavage (BAL) and biopsy was performed, dropping the patient’s oxygen saturation to 80% post-EBUS with an increased respiratory rate of 20–40 bpm. The patient was given two breathing treatments and was placed on 10–15 L of oxygen through high flow nasal cannula (HFNC) during transfer to the intensive care unit (ICU) for hypoxia and hypercarbic respiratory failure. During the patient’s stay in the ICU, the patient was placed on furosemide, budesonide, ipratropium bromide/albuterol sulfate, cefepime, and vancomycin due to worsening hypoxia, noting that he received two more radiation sessions before release back to hospitalist. Prior to release, biopsy results confirmed the patient’s mediastinal mass as poorly differentiated sarcomatoid carcinoma, with immunohistochemical expression of CK7 and vimentin and cytologic features including spindle-shaped and multinucleated giant cells. Vascular surgery was consulted prior to release, concluding that there was no operative intervention for the patient at this time and that he should continue radiation therapy.

**Figure 4:**
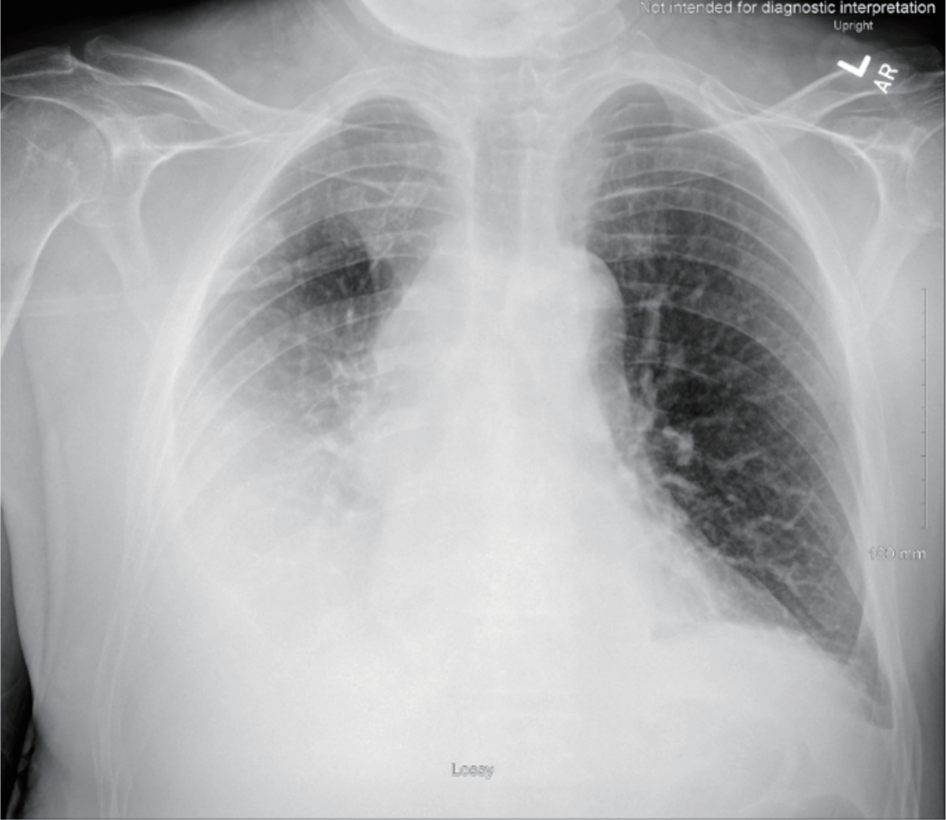
Chest X-ray obtained on hospital day 14, depicting an increase of right pleural effusion. Trace left pleural effusion and bibasilar areas of consolidation that may reflect atelectasis.

As the patient’s respiratory rate and SpO_2_ levels improved, he returned to inpatient services to complete three more radiation sessions. Even with the supplemental oxygen given, patient’s pleural effusion inevitably worsened (Fig. 4), prompting to multiple thoracenteses procedures and placement of the PleurX catheter. The patient later developed new-onset atrial fibrillation with rapid ventricular response (RVR), returning to the ICU where he was placed on amiodarone hydrochloride and apixaban. As the patient’s pericardial effusion worsened, a pericardiocentesis procedure was performed and biopsied for a malignant cytology confirmation. A pericardial window was not recommended per cardiothoracic surgery due to patient’s concerns of continuing radiation treatment, suspicions of atrial fibrillation secondary from the radiation therapy, and large tumor invading SVC.

Despite completing five sessions of palliative radiation therapy (total dose of 2,000 cGy, delivered as 400 cGy per fraction over 7 days), the patient’s condition continued to worsen, marked by persistent respiratory failure and functional decline. He was ultimately stabilized and discharged home on room air with a medication regimen that included amiodarone, apixaban, morphine sulfate, and oxycodone hydrochloride. Referrals were made to cardiology, oncology, and palliative care for ongoing multidisciplinary management.

Subsequent follow-up with cardiology and family medicine confirmed malignant cytology consistent with adenocarcinoma, characterized by gland-forming cells and positive thyroid transcription factor 1 (TTF-1) expression. Furthermore, at his outpatient oncology visit, a 10% programmed death-ligand 1 (PD-L1) expression was noted. Through shared decision-making, the patient decided against chemotherapy and immunotherapy, and opted for hospice care with an emphasis on comfort and quality time with family.

## Discussion

This case is unique in that the patient presented not only with a rare etiologic driver of SVC syndrome but also developed an extensive list of typical and atypical comorbidities: recurrent pleural effusions, hypoxic respiratory failure, pericardial metastasis, and new-onset atrial fibrillation. Furthermore, a follow-up diagnosis concluded the presence of adenocarcinoma from the pericardial effusion, demonstrating the diagnostic complexities of sarcomatoid histology.

Typically, SVC syndrome is diagnosed when an obstruction is directly compressing on or invading the SVC [2]. Similarly, this patient had a large mediastinal mass with severe SVC and right pulmonary artery compression, composed of a sarcomatoid carcinoma. SVC syndrome is mostly associated with malignancies such as small cell lung cancer (SCLC) and non-small cell lung cancer (NSCLC) [9]. While patients with SCLCs are more prone to develop SVC syndrome, the higher prevalence percentage in NSCLCs (80–85% of lung cancers) results in SVC syndrome being more common in NSCLCs [10]. NSCLCs can be broken down into three main epithelial carcinomas: adenocarcinoma, squamous cell carcinoma, and large cell (undifferentiated) carcinoma [11].

Specifically in this patient, due to the progression of the pericardial effusion, a pericardiocentesis was performed. This not only helped relieve the patient’s dyspnea but also confirm the presence of adenocarcinoma through the removed fluid. Adenocarcinoma is mostly seen in current smokers, former smokers, and non-smokers [11] (accounts for 40% of all lung cancers [12]), and metastasizes when glandular epithelial cells change/rapidly grow out of control [13]. Even though adenocarcinoma is the most common NSCLC, the most common cell type in SVC syndrome associated with NSCLCs is squamous cell lung cancer [14]. Squamous cell carcinoma metastasizes through squamous cells (accounts for 30% of all NSCLCs [15]), flat cells found near a main airway [11], and are often associated with smoking [11]. Moreover, large cell (undifferentiated) carcinoma can be found in any part of the lung, and accounts for around 9% of all lung cancers [16].

Uncommonly, lung cancer patients may encounter sarcomatoid carcinoma, accounting for approximately 0.1% to 0.4% of reported pulmonary tumors [17]. Pulmonary sarcomatoid carcinoma (PSC) can be broken down into five separate groupings: pleomorphic carcinoma (PC), spindle-cell carcinoma (SCC), giant-cell carcinoma (GCC), carcinosarcoma, and pulmonary blastoma [18]. PCs, accounting for upward of 50% of all PSCs, are composed of sarcomatoid characteristics (such as spindle and giant cells) with a potential combination of epithelial carcinomas [18]. Moreover, SCCs are mainly composed of malignant spindle cells, GCCs are composed of large pleomorphic tumor cells oriented in a sarcoma-like pattern, carcinosarcomas are composed of adenocarcinoma or squamous cell carcinoma with either malignant fibrohistiocytoma or fibrosarcoma, and pulmonary blastomas are comprised of primitive epithelial elements (resembling well-differentiated fetal adenocarcinoma, along with a mesenchymal stroma containing blastemal-like cells) [18].

Genetically, mutations in the epidermal growth factor receptor (EGFR), anaplastic lymphoma kinase (ALK), and Kirsten rat sarcoma virus (KRAS) have seen to be associated with the development of NSCLC [19]. Similarly, PSCs are commonly associated with EGFR and ALK mutations [18]. Furthermore, cytokeratin, a specific epithelial marker present in most NSCLCs [20], remains consistent during malignant transformation of 79% of PSCs (stained with pan-cytokeratin) [21]. Due to its poorly differentiated nature and rapid progression, PSC is often diagnosed at very advanced stages, resulting in a poor prognosis with limited therapeutic options [22]. In addition, due to the low prevalence of PSC [17], minimal studies have focused on the association between PSC and SVC syndrome.

Management of SVC syndrome secondary to malignancy is typically guided by the severity of the patient’s symptoms and the underlying pathology of the case. Current guidelines recommend a multi-level approach based on severity: steroids, head elevation, and use of supplemental oxygen for symptoms relief; radiation/chemotherapy to reduce mediastinal mass; and endovascular stenting for cases with severe symptoms such as oropharyngeal or cerebral edema [4]. In this case, the patient underwent a total of five urgent palliative radiation therapy sessions, in attempt to reduce the size of the mediastinal mass. Endovascular repair was not pursued by cardiothoracic surgery or vascular surgery in this case due to the tumor’s extensive invasion and poor surgical candidacy. This approach was consistent with guideline-based practice, with the plan of action being decided based on the severity of the mediastinal mass as well as the patient’s overall symptomatic status.

### Conclusion

This case highlights a rare co-occurrence between SVC syndrome secondary to PSC. The patient’s clinical workup was complicated by recurrent pleural effusions, pericardial effusions, hypoxic respiratory failure, and new-onset atrial fibrillation with RVR, emphasizing the severity and rapid progression of SVC syndrome. These complications prompted ICU-level care, invasive procedures (pericardiocentesis, PleurX catheter), and high-dose antiarrhythmic therapy. Optimizing patient care required consultation and coordination between oncology, family medicine, cardiothoracic surgery, pulmonology, cardiology, and palliative care. Early hospice integration, supporting the patient’s comfort and values, highlights the significance of shared decision making in an advanced malignancy. Clinicians should maintain a high index of suspicion for SVC syndrome in patients with progressive upper body swelling and respiratory symptoms, as early recognition and coordinated care are essential for optimizing outcomes.

## Data Availability

The data supporting the findings of this study are available from the corresponding author upon reasonable request. ALK, anaplastic lymphoma kinase; BAL, bronchoalveolar lavage; CAP, community-acquired pneumonia; COPD, chronic obstructive pulmonary disease; CT, computed tomography; CTA, computed tomography angiography; DVT, deep vein thrombosis; EBUS, endobronchial ultrasound; EGFR, estimated glomerular filtration rate; GCC, giant-cell carcinoma; HFNC, high-flow nasal cannula; ICD, implantable cardioverter-defibrillator; ICU, intensive care unit; KRAS, Kirsten rat sarcoma virus; MRV, magnetic resonance venography; NSCLC, non-small cell lung cancer; PC, pleomorphic carcinoma; PD-L1, programmed death-ligand 1; PSC, pulmonary sarcomatoid carcinoma; RVR, rapid ventricular response; SCC, spindle-cell carcinoma; SCLC, small cell lung cancer; SVC, superior vena cava; SVT, supraventricular tachycardia; VTE, venous thromboembolism; WBC, white blood cell count
